# Experiences of Loss and Grief Among Brazilian Frontline Healthcare Professionals During the COVID-19 Pandemic Crisis: A Grounded Theory Analysis

**DOI:** 10.3390/healthcare14091230

**Published:** 2026-05-03

**Authors:** Paola Kallyanna Guarneri Carvalho de Lima, Carlos Laranjeira, Amira Mohammed Ali, Feten Fekih-Romdhane, Murat Yıldırım, Lígia Carreira, Maria Aparecida Salci

**Affiliations:** 1 Department of Postgraduate Nursing, State University of Maringá, Avenida Colombo, 5790-Campus Universitário, Maringá 87020-900, Brazil; paolakgcl@gmail.com (P.K.G.C.d.L.); ligiacarreira.uem@gmail.com (L.C.); masalci@uem.br (M.A.S.); 2 School of Health Sciences, Polytechnic of Leiria, Campus 2, Morro do Lena, Alto do Vieiro, Apartado 4137, 2411-901 Leiria, Portugal; 3 Centre for Innovative Care and Health Technology (ciTechCare), Polytechnic University of Leiria, Campus 5, Rua das Olhalvas, 2414-016 Leiria, Portugal; 4 Comprehensive Health Research Centre (CHRC), University of Évora, 7000-801 Évora, Portugal; 5 Department of Psychiatric Nursing and Mental Health, Faculty of Nursing, Alexandria University, Smouha, Alexandria 21527, Egypt; amiramali@alexu.edu.eg; 6 Faculty of Medicine of Tunis, Tunis El Manar University, Tunis 1007, Tunisia; feten.fekih@gmail.com; 7 The Tunisian Center of Early Intervention in Psychosis, Department of Psychiatry, Ibn Omrane, Razi Hospital, Manouba 2010, Tunisia; 8 Department of Psychology, Faculty of Science and Letters, Ağrı İbrahim Çeçen University, Ağrı 04100, Türkiye; muratyildirim@agri.edu.tr; 9 Psychology Research Center, Khazar University, Baku AZ1000, Azerbaijan

**Keywords:** COVID-19, grounded theory, grief, loss, healthcare professionals, Brazil

## Abstract

**Background/Objectives**: The COVID-19 pandemic exerted unprecedented pressure on healthcare professionals and systems worldwide. To manage this increased demand, hospitals extended working hours, resulting in increased strain on workers and impacting their professional well-being. Simultaneously, the numerous deaths due to illness meant that healthcare professionals did not have sufficient time to process grief, which may have led to unresolved grief and other mental health problems. The aim of this study was to understand the experiences of loss and grief and their repercussions on Brazilian healthcare professionals working on the front lines during the COVID-19 pandemic. **Methods**: This qualitative study followed Charmaz’s constructivist grounded theory. The study used the COREQ checklist. Between August 2024 and January 2025, 24 healthcare professionals who worked on the front lines during the COVID-19 pandemic were interviewed via telephone. Participants were primarily female (n = 14) with a mean age of 42 years (SD = 9.13). Interviews were audio-recorded and transcribed. **Results**: The core phenomenon that emerged from the analytical process is “between exhaustion and resilience in a war-like scenario: challenges and opportunities in the care provided by frontline professionals during the COVID-19 pandemic”. This main axis was anchored in three categories: (1) adversities imposed by COVID-19 on the functioning of health services; (2) witnessing sudden deaths and the physical absence of families; (3) reconstruction of meanings and personal and professional growth. **Conclusions**: The experience of grief was intensified by the peculiarities permeating the death process in the pandemic context and the modification of farewell moments. The study exposes a need for training programs focused on medical, nursing, psychological, and other areas of care education that involve not only understanding clinical issues but also recognizing loss and grief as an integral part of care processes. Importantly, legislators should allocate additional resources to services that provide psychological support to healthcare professionals, in order to promote their adaptive coping.

## 1. Introduction

COVID-19 was declared an international pandemic by the World Health Organization on 11 March 2020. The world suffered countless deaths and infections; by January 2026, more than 779 million infections and seven million deaths had been recorded [1]. The pandemic placed unprecedented pressure on health systems. The capacity of health systems in many regions was surpassed by the high volume of cases, hospitalizations, and deaths. In response to this heightened demand, hospitals prolonged staff work hours, decreased vacation time, and expanded their intensive care units [2,3].

The pandemic brought about significant changes in the functioning of health systems globally with a direct impact on work practices and the occupational scope of health professionals [4,5,6,7,8]. Throughout the different COVID-19 pandemic waves, health professionals worked long hours, under high levels of pressure, and had to deal with traumatic deaths and significant ethical dilemmas in decision-making [9,10]. In addition, changes in health system functioning varied during the pandemic, depending on factors such as the pathogen’s characteristics, transmission dynamics, severity, duration, available treatments or vaccines, and the sociopolitical and economic context [11].

Healthcare professionals experienced tension and moral distress due to the virus’ unpredictability, uncertainty about care provision, feelings of helplessness and fear, isolation from family, and the rapid dissemination of misinformation [12,13]. Evidence suggests that healthcare professionals were especially vulnerable due to the psychological stressors faced during the pandemic. Joint estimates indicate that 28.5% of professionals experienced clinically significant symptoms of depression, 28.7% suffered from anxiety, 25.5% developed post-traumatic stress disorder, and 24.4% faced insomnia [14], resulting in reduced levels of empathy [15].

Mental health problems, including anxiety, depression and post-traumatic stress, disproportionately affected healthcare professionals who were younger, had less work experience and heavier workloads, worked in unsafe environments, and those lacking adequate training and social and psychological support [16]. Working on the front lines caused not only psychological stress but also significant changes in work environments [17], especially in specific COVID-19 units and emergency departments [18]. Burnout, job abandonment [19], and compassion fatigue [20] were some of the work-related phenomena that emerged, especially due to the absence of psychological counseling and occupational health services [21].

During the COVID-19 pandemic, there was a concomitant increase in job turnover rates [22,23]. In most situations, professionals manifested psychological responses to negative aspects of the job, and in extreme situations, they decided to leave the profession [24]. Another highly prevalent phenomenon was dealing with numerous deaths in a short period of time, without enough time to process grief, generating inhibited or unresolved grief [25,26]. Feeling a sense of responsibility and obligation to protect patients’ lives may also have increased the risk of prolonged grief [27].

Grief is a healthy process that helps adapt to loss. However, responding to multiple deaths during the COVID-19 pandemic posed a significant challenge for healthcare professionals [28,29,30,31]. The unique bond between healthcare professionals and patients gives rise to professional grief, which is often not recognized or accepted by the general public [32,33]. In the context of healthcare, professional grief is the emotional distress experienced by healthcare professionals after the death of patients during clinical practice [34,35]. Symptoms commonly associated with this distress include sadness, self-blame, sleeplessness, reduced appetite, and recurring thoughts of death [33].

In sum, frontline healthcare professionals faced multiple risks during the pandemic, whether from constant exposure to SARS-CoV-2 infection, or from long working hours, burnout, stigma, exhaustion, violence, and the risk of developing mental disorders [36,37]. In this sense, maintaining the health and safety of this workforce was crucial to responding to future health emergencies and providing quality, person- and family-centered care [38]. If staff well-being is not maintained, the ability of the health system to provide quality care to patients will be at risk [39,40].

Some healthcare professionals may ignore feelings of grief and use strategies, such as avoidance or submission, to continue working [41,42]. However, an inadequate or incomplete grieving process can result in pathological or complicated grief, leading to dysfunctions in personal and professional life [43]. The grieving process takes time to heal, and family and social support are facilitating elements [44]. Addressing grief and loss among healthcare professionals in post-pandemic Brazil is essential, given the high exposure to death, moral distress, and systemic vulnerabilities experienced during COVID-19 [45,46]. Recognizing and supporting these experiences is crucial for individual well-being, workforce sustainability, and the quality and humanization of healthcare delivery [45,46].

Given this scenario, the following research question arose: What were the experiences of loss and grief and their repercussions on frontline healthcare professionals during the COVID-19 pandemic? This study aimed to understand the experiences of loss and grief and their repercussions on frontline healthcare professionals during the COVID-19 pandemic in the Brazilian context. It is hoped that this study can support interventions to maintain the health and well-being of healthcare professionals after the COVID-19 pandemic and provide better preparedness for future pandemic scenarios.

## 2. Materials and Methods

### 2.1. Study Design

This qualitative study used a constructivist grounded theory (CGT) approach, as developed by Charmaz [47]. Qualitative methods were deemed the most suitable for capturing human experiences within their social context [48], refusing to see the world as an objective reality [47]. Rather, it adopts a social constructivist viewpoint where various interpretations of reality can exist simultaneously and be jointly developed by the researcher and the participant. The research was guided by the Consolidated Criteria for Reporting Qualitative Research (COREQ) checklist [49,50].

### 2.2. Setting, Participants and Recruitment

This study was conducted in the state of Paraná, in the southern region of Brazil. Paraná is the 5th state in number of deaths from COVID-19. According to data from the Coronavírus Brasil portal, from the beginning of the pandemic until September 2025, there were 3,041,284 million cases, with 47,082 deaths from the disease in the state [51].

To answer the goals of the current study, the following eligibility criteria were used: (a) being 18 years of age or older; (b) being a nursing, medical, or psychology professional who worked on the front lines during COVID-19 (i.e., worked in close contact with other people during the pandemic); (c) being from the state of Paraná; (d) having the ability to communicate and understand Portuguese; and (e) having responded to the telephone contact (up to three attempts). All participants were recruited regardless of sex, race, and seniority to ensure maximum sample variation. For participant recruitment, a mixed approach was used to select participants who met the established criteria through a chain of referrals [47,52]. Initially, contact was made purposively with a nurse who worked on the front lines during COVID-19, who then identified other healthcare professionals who worked during the required period (snowball sampling technique), thereby informing the research design. The option to include different professionals (e.g., physicians, nurses and psychologists) is aligned with previous evidence indicating that repeated exposure to patient deaths generated emotional distress across these roles, regardless of specific profession [53,54].

Throughout the research, the sample size underwent continuous assessment to guarantee it yielded adequate data for the study’s objectives, ultimately reaching theoretical data saturation. This strategy enabled the researcher to assess the necessary number of interviews to achieve the purpose of the research, based on the level of analysis and the datasets emerging from the interviews [55,56]. Once theoretical saturation was reached and no further insights were derived from the data, sampling was terminated [47]. The final sample consisted of 24 healthcare professionals.

### 2.3. Data Collection

Data was collected between August 2024 and January 2025. Eligible participants were contacted by telephone. In the initial contact, the study objectives and the importance of participating were explained. After acceptance, participants were sent the Informed Consent Form through their preferred channel (WhatsApp or Email), and the day and time of the interview was mutually agreed upon. Data collection was carried out by a female doctoral student in nursing (P.L.) with approximately seven years of clinical experience and skills in conducting qualitative research.

The interviews followed a semi-structured script, developed according to the study’s objectives and informed by the available literature [57,58,59]. The interview guide was validated by three doctoral researchers with experience in the subject, with minor adjustments to the wording of the questions. The guide was designed to gather information about work contexts during the pandemic, with a special emphasis on experiences of loss and professional bereavement (Appendix A). Each interviewee was first asked, “What was it like for you to work on the front lines during the COVID-19 pandemic?” Follow-up questions were used to explore and clarify the topic: “What was it like dealing with death and bereavement?”; “Tell me more about that”; “Can you give me an example?”. The interviews lasted between 30 and 50 min (average of 40 min) and were audio-recorded on a digital voice recorder. There were no repeated or follow-up interviews.

### 2.4. Data Analysis

After the data collection phase, participant interviews were transcribed using the Transkriptor tool, and the data were managed, stored, and analyzed using the MaxQDA^®^ 24 software [60]. After interview transcription, the analysis consisted of reading and understanding all the interviews, aiming to better categorize them by thematic similarity according to the questions asked and the answers given by the participants throughout the interview. In the end, the data obtained were classified into categories and subcategories according to the health professional’s experiences of loss and grief during the COVID-19 pandemic. Adopting a constant comparative analysis approach, data collection and analysis occurred simultaneously. Coding was completed after each interview. Three levels of coding were used: (I) an initial coding where identifying words, lines, or segments were identified to help researcher become acquainted with the data and uncover early conceptual ideas; (II) a focused coding analyzing extensive data by grouping codes systematically in search for explanation and meaning; and (III) a theoretical coding through memo writing/elaborated categories [47,55]. The main investigator led the coding and analysis process and discussed it in regular team meetings. All members of the research team reviewed and agreed on the final codes and categories.

### 2.5. Rigor

Charmaz and Thornberg [61] proposed four criteria to ensure the rigor and validity of research: credibility, originality, resonance, and usefulness. Credibility was obtained through a set of strategies: reflexivity, prolonged engagement, and peer debriefing [61]. The interview technique, in addition to being a means of data collection, was used as a tool for active listening, serving to promote, broaden, and deepen the contact between the researcher and the participants. In this process, the researcher was challenged to explicitly state their premises and assumptions [61]. Based on this assertion, the first author (P.K.G.C.L.) is a doctoral candidate in nursing, with clinical experience working with bereaved individuals. The second author (C.L.) is a nursing faculty member with extensive experience in guiding and developing grounded theory studies. He supervised all stages of the research process. The other authors are associate professors with prior experience in conducting qualitative studies and analyzing qualitative data. The second criterion—originality of findings—was established through a reflective process conducted with the project team members, involving the writing of memos and the evaluation of existing literature. Resonance was achieved by a combination of deductive and inductive analysis as fostered by the CGT method, along with a schematic representation of the phenomenon. Lastly, utility was satisfied by developing the participants’ understanding of their daily experiences and by providing a foundation for the development of good practices applicable in similar contexts.

### 2.6. Ethical Considerations

The study protocol was approved by the Research Ethics Committee of the State University of Maringá-UEM (Opinion No. 4.214.589). Each participant gave informed consents, including permission for audio recording. Participants were informed they could withdraw from the study at any time. There was no financial compensation for participation. In response to participant distress, the interviewer would offer immediate assistance and direct participants to professional bereavement support services. To preserve participant identity throughout the discussion, they were identified by the word “participant,” followed by their age (e.g., P1, 21 years).

## 3. Results

### 3.1. Participants Background

The study included 24 healthcare professionals, mostly female (n = 14), aged between 25 and 59 years (42 ± 9.13), and white (n = 22). Regarding education, most participants have postgraduate training (n = 19; 79.2%). All research participants worked on the front lines during the COVID-19 pandemic. Some participants worked in more than one sector, the most common sector being hospital inpatient units (n = 11), followed by Intensive Care Units (n = 10), and other sectors including Primary Health Care Units (n = 3) (Table 1).

### 3.2. Findings Overview

**Figure 1:**
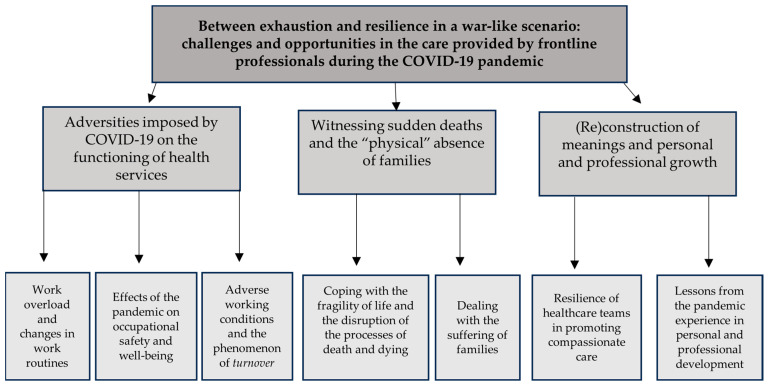
Categories and subcategories of the phenomenon “Between exhaustion and resilience in a war-like scenario: challenges and opportunities in assisting frontline professionals during the COVID-19 pandemic”.

The analytical process led to the central phenomenon: “Between exhaustion and resilience in a war-like scenario: challenges and opportunities in the care provided by frontline professionals during the COVID-19 pandemic”. This main axis was anchored in three categories and seven subcategories that support the phenomenon and help understand the findings (Figure 1). The three categories identified were: (1) adversities imposed by COVID-19 on the functioning of health services; (2) witnessing sudden deaths and the “physical” absence of families; and (3) (re)construction of meanings and personal and professional growth. The categories were sequentially related to illustrate a process in which systemic adversities shaped repeated exposure to death and relational rupture, prompting healthcare professionals to engage in meaning-making processes that resulted in personal and professional growth. The complexity of the work associated with the pandemic brought numerous challenges to the care provided by frontline healthcare professionals. Being available to accommodate the subjective expressions of patients and families generated tension, requiring professionals to deal with their own subjectivity, their own grief, or even feelings of inadequacy.

#### 3.2.1. Adversities Imposed by COVID-19 on the Functioning of Health Services

The first category has three subcategories. The first subcategory is work overload and changes in work routines, which reflects the multifaceted impact the COVID-19 pandemic had on health professionals. Most professionals reported that the pandemic experience was chaotic, given the lack of guidance on how to deal with the situation and the difficult management of hospital beds in the face of the high volume of infection cases.

*[…] In the beginning, everything was very crazy because nobody knew anything, and there was so much despair. There wasn’t much information yet on how to enter the ward, how to treat patients. I saw professionals going crazy, even mistreating the residents, due to the uncertainties*

(P8, 28 years old).

*[…] In the beginning, there wasn’t a protocol for treatment or anything, because until then we didn’t know what it was all about. I didn’t know what to do. Everyone was kind of lost, right? There was no way to cope*

(P2, 32 years old).

*[…] Having people ask for help, in an entire hospital, and us not having enough answers. We had to refuse patients because we couldn’t receive them*

(P1, 33 years old)

Excessive working hours, the absence of days off, and the need to replace patient colleagues led to a permanent state of exhaustion and fatigue, aggravated by rapid changes in patient health status and the anxiety generated by unpredictable prognoses.

*[…] Colleagues also got sick, because there was no way around it, we would get infected, even if we protected ourselves. We worked with a lot of patients, with a reduced team. They were leaving or getting sick*

(P2, 32 years old).

*[…] It was a stage that no healthcare professional will ever forget. Never again, because we worked hard. I would go in at 8 am and leave at 5 pm from the inpatient ward. Then, from 6 pm to midnight, I would go to the COVID Care Center. It was a difficult time for everyone*

(P22, 48 years old).

*[…] In fact, we were used to seriously ill patients, but we started having patients who deteriorated very quickly, and we lost those patients. We were very anxious*

(P5, 31 years old).

Furthermore, social distancing, difficulties in visiting family, and changes in personal life, coupled with a lack of psychological support, resulted in high levels of emotional overload. Inadequate compensation and a lack of recognition from society and public policies devalued the work of these professionals. The constant worry about transmitting the virus—both becoming infected and transmitting it to family members, colleagues, or other patients—intensified stress and fear.

*[…] I wasn’t going to see my parents, I was suffering here because I’m an only child, I’m very attached to them, one day I found out they tested positive, I got in the car and went to their city, a two-hour drive, and I stayed five minutes at the gate, just to see them. And I came back home because I was so desperate, but I needed to see, because I just cried, for me that was a death sentence for them, and they were okay, we lost a lot of people in the ICU, it was the worst time, there was no vaccine yet, so it was desperate*

(P8, 28 years old).

*[…] I went months without seeing my family, I only talked on the phone, my mother and my aunt, they would come to my house, leave food for me at the gate, I was on duty, but we didn’t have that kind of contact for a long time*

(P11, 32 years old).

The second subcategory—effects of the pandemic on occupational safety and well-being—reflects the complexity of the experiences of frontline healthcare professionals, due to the intertwining of structural, psycho-emotional, and ethical factors. Professional burnout was not limited to workload, but involved moral suffering, lack of adequate support, and the constant struggle to balance professional demands with personal and family needs.

*[…] In the beginning, what happened was that he had no initiative or support, there was no conversation or dialogue. He didn’t even have a cell phone for video calls with family members; the basics no longer existed. It took a long time for the hospital to organize itself around this, let alone support beyond the basics, which would be emotional support, something like that, you know*

(P21, 29 years old).

*[…] I kept going over the PPE I had to put on, so that somehow, it would reassure me that I would at least be protected, you know? And I would go over that mechanism before entering the room so that once inside I would also feel…capable of helping that person. Because if I focused on the risk I was in, I wouldn’t be able to attend to that person in a humanized way*

(P10, 32 years old).

The third subcategory—adverse working conditions and the phenomenon of turnover—explores how the pandemic not only demanded the total dedication of healthcare professionals but also pushed many to the limit of their physical, emotional, and mental capabilities, resulting in decisions that profoundly transformed their life trajectories. Many faced such a severe level of burnout that it resulted in radical changes, such as the decision to leave the profession, move to another city, or completely reshape their routine.

*[…] At the institution where I worked, many people also requested to leave due to the workload overload, which led them to give up the profession because the working conditions were no longer adequate. And we were also able to observe this because many people left nursing, the workload increased, there was no adjustment, and there was also this issue of instability on the part of the hospital institutions*

(P4, 28 years old).

*[…] We had no support, so much so that I got sick at the hospital and ended up quitting. I earned very well at the hospital because we had a salary bonus due to COVID, but there was no psychological support; on the contrary, there was a lot of pressure from management*

(P12, 25 years old).

*[…] We, the healthcare professionals, are much more important than we imagine, especially nurses. It was a time when we worked a lot, but we weren’t recognized enough, and we ended up being forgotten*

(P6, 32 years old).

The COVID-19 pandemic imposed extreme challenges on healthcare professionals, impacting not only their work schedules but also their personal lives and career prospects. Despite the temporary recognition of their efforts, many professionals reported a feeling of devaluation once the crisis was under control, which contributed to a feeling of frustration and abandonment. For some, the only solution was to step away, even though they loved their profession.

#### 3.2.2. Witnessing Sudden Deaths and the “Physical” Absence of Families

The second category addresses the intense experiences of loss and grief experienced by healthcare professionals during the COVID-19 pandemic, highlighting the emotional and moral impact generated by constant contact with death and the suffering of patients. The isolation of patients at critical moments, such as the end of life, and the forced separation from family members intensified the moral suffering of these professionals, who often felt powerless in the face of the restrictions imposed by health safety measures.

The first subcategory—coping with the fragility of life and the disruption of the processes of death and dying—demonstrates how healthcare professionals experienced the rapid and sudden death of patients, often young and involving several members of the same family, making death a constant presence and generating a strong emotional impact.

*[…] I saw people who died on the same day, sometimes young people, without pre-existing illnesses, without comorbidities, without anything, dying. So it was something that messed with my head a lot. After that time, I developed anxiety, it turned into a difficult disease to treat*

(P4, 28 years old).

*[…] It was very impactful, one night six people died, just in my ICU*

(P7, 54 years old).

*[…] And it was always very painful having to communicate with the family and having to call them to identify the body, to have those ten minutes there, which was the time we could spend with them there, right?*

(P15, 27 years old).

The participants’ accounts highlight attempts to offer some final comfort to family members, but these attempts did not always fulfil the need for farewell due to the absence of in-person rituals such as touch or hugs, which further aggravated the difficulty of the grieving process. These elements reveal the depth of the suffering experienced, the ethical dilemmas faced, and the lasting impact of these experiences on the practice and mental health of healthcare professionals.

The second subcategory—dealing with the suffering of families—reflects positive aspects emerging from the experience of health professionals during the pandemic, highlighting the redefinition of care in the face of a challenging scenario. Despite the suffering and losses, many professionals reported a strengthening of empathy, compassion, and sensitivity, improving the quality of care provided. The possibility of providing comfort in the last moments of life, facilitating virtual farewells, and offering psychological support proved to be an essential practice in the humanization of care.

*[…] We have to be compassionate, driven by the desire to help people. It is the personal commitment we have to the profession*

(P21, 29 years old).

*[…] What we valued was providing farewells to the family, even if only for brief moments*

(P15, 27 years old).

*[…] What truly made the difference, even if for five minutes, was providing the last goodbye, that last look, that recognition. In this way, the family was able to better process what was happening*

(P16, 35 years old).

The experience of dealing with striking family stories, such as couples who passed away without knowing of each other’s departure, or witnessing recoveries considered miraculous, reinforced the perception of the value of person-centered care. In addition, addressing the spiritual side and a deeper understanding of the death process contributed to a transformative learning experience.

*[…] There were many couples who were hospitalized at the same time, so we organized ourselves to keep them together. There was a specific case where they started arguing, each complaining about the other, and we decided to separate them. After the separation, the husband began to worsen, he went to the ICU. The wife stayed in the ward without knowing about her husband, because the children asked us not to tell her. Two days later the wife also worsened, and both ended up passing away, they were left without knowing about each other… these are family stories that mark us*

(P12, 25 years old).

*[…] There was a medical student with COVID, he worsened and we thought he wouldn’t survive. But he survived, he was a miracle, I saw some miracles. And he left, and on the day he left, we lined up and took a picture; it was even published on Globo TV. It was moving to know that we were able to help some people, providing dignity in their care*

(P7, 54 years old).

The pandemic experience promoted a significant change in how these professionals viewed their care practice, prioritizing listening, respect for individuality, and offering comfort, even in farewell situations. Thus, this category demonstrates how the pandemic, despite its burden of pain, also enabled the development of a more humanized professional practice, based on empathy, understanding of others, and valuing human dignity.

#### 3.2.3. (Re)Construction of Meanings and Personal and Professional Growth

This category explores how the pandemic, despite its difficulties, generated a profound transformation in healthcare professionals, making them more resilient, aware of their role, and prepared for future challenges. The first subcategory—resilience of healthcare teams in promoting compassionate care—indicates the pandemic provided not only challenges but also opportunities for growth and maturation of professionals.

*[…] At the time, it was one day at a time, trying to survive. We saw so many young people fighting until the last second to live. Only now have I been able to stop, to give myself this opportunity to live too*

(P8, 28 years old).

*[…] I think the first lesson I learned was to take care of myself, to take care of others. The virus changed our entire routine and our way of thinking. We had the chance to give new meaning and completely change our lives*

(P17, 33 years old).

*[…] We learn a lot in life, we learn the value of life. Often, people only work and don’t pay attention to their families, to their young children who need it. We saw so many people dying and leaving their families behind, so much suffering. We ended up becoming more attached to our families, wanting to offer words of comfort to those who lost friends and family. So, it was a huge learning experience*

(P22, 48 years old).

Furthermore, contact with death and human fragility led to greater emotional resilience. Psychological growth was a central element, leading professionals to invest in self-care, as well as learning to block unnecessary emotions to preserve their energy. The strengthening of bonds, both with colleagues and with patients and family members, highlighted the centrality of human relationships in the profession.

The second subcategory—lessons from the pandemic experience in personal and professional development—highlights the positive aspects experienced by healthcare professionals during the COVID-19 pandemic, emphasizing how, even amidst extreme challenges, there were opportunities for learning, and strengthening of professional bonds. The experience gained in techniques and procedures, combined with the adaptation and standardization of practices in the face of the crisis, provided significant professional maturity.

*[…] I think it was one of the greatest experiences I’ve had in my life, right? Even in such a painful, suffering, and chaotic context, it was a time of great learning. We helped set up the new ICU in terms of equipment, bed setup, structure, and protocols*

(P15, 27 years old).

*[…] Having lived through a pandemic brought about the strengthening of interpersonal relationships within the teams*

(P10, 32 years old).

*[…] COVID brought a great deal of experience, above all in the ability to deal with human suffering*

(P5, 31 years old).

Solidarity and unity among multidisciplinary teams were fundamental to strengthen the collaborative spirit and create support and cooperation networks. The pandemic also accelerated the growth of cooperative tools such as telecare, in addition to boosting investments in digital health, expanding the capacity for care and innovation.

*[…] Everyone working together, that was the most beautiful thing about the pandemic. Everyone working together for a moment, the nurses, the doctors, the physiotherapists, the psychologists, it was beautiful to see*

(P8, 28 years old).

*[…] It was a scene of terror, but it was where we saw all the hospitals coming together, everyone uniting. So, that was beautiful to see*

(P13, 29 years old).

*[…] It showed the need to have telecare as well, to develop digital health, we didn’t have any of that*

(P7, 54 years old).

## 4. Discussion

This study explored the experiences of loss and grief and their repercussions among frontline healthcare professionals during the COVID-19 pandemic in Brazil. Based on a set of interviews conducted with healthcare professionals who worked on the front lines during the COVID-19 pandemic, it was possible to identify the core category “Between exhaustion and resilience in a war-like scenario: challenges and opportunities in the care provided by frontline professionals during the COVID-19 pandemic”. The use of constant comparative analysis and coding techniques allowed us to acquire a rich and well-founded understanding of these health professionals’ views on their work situation and their experiences of loss and grief as a result of COVID-19.

The narratives of health practitioners provide valuable insights into the diverse forms of loss they encountered in their clinical duties. They also reveal the severe repercussions these losses had on both their personal and professional lives, especially given the inadequacy of institutional acknowledgement and assistance [41,62,63]. The accumulated experience of numerous patient deaths has long-lasting personal and professional effects on health workers [32]. In this context, coping with the grief of professionals involves strategies like (re)building the meaning of death, developing self-adjustment behaviors, and searching for support [57].

Healthcare professionals faced difficulties in finding time to process grief and loss caused or intensified by the COVID-19 pandemic. While assuming unprecedented heavy workloads, they faced numerous losses in their work environments that often projected into their personal lives, increasing their levels of exhaustion, overload, and burnout [31,57,64]. Another relevant factor reported was the difficulty in communication between professionals and patients, and between patients and their families. Indeed, deficient or absent communication increases the suffering perceived by those bereaved by COVID-19 [65,66,67]. In parallel, one of the most reported fears among healthcare professionals during the pandemic was that of becoming infected or infecting others, particularly their family members [68]. A high perceived risk of infection has been associated with anxiety, distress, insomnia, social dysfunction, and depression [69,70]. Many of these symptoms were reported by this study’s participants.

Medical staff personally observed and experienced loss and grief, and their collective suffering reflects the lack of “spaces” to process their loss. The pandemic disrupted numerous social mechanisms linked to the ritualization of death and grief, which are essential for reducing suffering and promoting the welfare of the bereaved [31,71].

People were prevented from saying goodbye in the customary manner because they couldn’t be with their loved ones in their final moments. The disruption of mourning rites hindered the ability of bereaved individuals to find closure and begin healing. Healthcare professionals witnessed and experienced this disruption of the grieving process. They were frequently the last person to be with all those who died throughout the pandemic, without their loved ones and family members. Although end-of-life care was very rudimentary due to the catastrophic scenario, it included actions that brought comfort to those who were dying, promoting relational dignity and listening to their interests, motivations, and needs [12,14,72]. While grappling with diminished health and job satisfaction, healthcare professionals admirably confronted the many challenges of the pandemic, particularly those concerning mental health [73,74]. This struggle caused emotional and psychological stress and exhaustion, harming their well-being in both personal and professional spheres. In this sense, many professionals expressed a desire to leave the profession (turnover), reinforcing the global shortage of healthcare professionals and threatening the efforts of healthcare organizations to provide safe and quality care [74,75].

Moreover, the ability to process the loss and suffering witnessed and experienced by professionals was significantly impacted by isolation—both from community life and from spaces of personal meaning. This social distancing also meant that these professionals often carried, alone, the burdens related to their responsibilities.

### 4.1. Strengths and Limitations

This study used qualitative interviews to assess participants’ real experiences, thus producing a deeper level of information. The CGT approach was chosen for this study because of its purpose of providing empirical knowledge in situations where prior hypotheses do not exist. This method explores and develops concepts from the collected data, rather than from prior assumptions. Research questions are addressed through open-ended exploration, wherein new perceptions and understandings emerge from the data.

However, there were several limitations in this study. First, the absence of visual cues via telephone interviews represented a risk of bias due to the loss of information related to nonverbal communication and may compromise rapport, probing, and interpretation of responses. Telephone calls did provide access to participants from the different parts of the State of Paraná. But transferring the results to other Brazilian states where COVID-19 had different impacts is limited. Likewise, as with most qualitative studies, the findings are context-specific and shaped by the sociocultural, organizational, and health-system characteristics of Brazil. Second, the cross-sectional nature of qualitative designs captures the experiences of loss and grief at a single point in time, limiting insight into the temporal evolution of grief. Among healthcare professionals, grief is likely to be dynamic, cumulative, and non-linear, particularly in the context of prolonged crises such as the COVID-19 pandemic. Consequently, this design does not allow the exploration of how grief reactions may transform, intensify, or attenuate over time, nor how professionals integrate loss into their personal and professional identities in the longer term. Indeed, there is a need to examine how grief and loss among healthcare professionals evolve over time, identifying patterns of adaptation, prolonged grief, moral injury, or post-traumatic growth in the post-pandemic period. Furthermore, the sample population was predominantly female and identified as white; therefore, experiences may differ from those of other professional groups with different gender and racial balances. In a racially heterogeneous country such as Brazil, where structural racism influences occupational exposure, vulnerability, and access to support [76], these experiences may differ significantly across groups. Moreover, participation was voluntary, which may have introduced self-selection bias, potentially favoring professionals who were more willing or able to articulate their experiences of grief. Also, participants’ recollections of past experiences may be subject to memory bias. Finally, the data analysis neglected variations in experiences arising from the severity of the various restrictions associated with the pandemic. Future studies should continue to understand the impacts of the pandemic on occupational grief in order to anticipate responses to new crisis events. Further research should identify individual, relational, and organizational factors that exacerbate or mitigate grief responses, including team support, leadership practices, institutional recognition, and access to mental health resources. Lastly, evaluative studies assessing the effectiveness of grief-informed interventions (such as reflective spaces, peer support programs, supervision models, and institutional rituals of remembrance) are needed, particularly within resource-constrained health systems.

### 4.2. Implications for Practice

Reflecting on the impact of loss and grief in the context of healthcare is crucial for the training and practice of professionals in the field. Medical, nursing, psychological, and other care education should involve not only understanding clinical issues but also recognizing and accepting the personal losses of the professionals themselves. Experiencing these losses directly influences how these professionals deal with grief [77,78].

Furthermore, professionals must be able to reflect on how the rules, goals, and organizational culture of their work environment can influence their behavior and emotional response in situations of loss. In this sense, healthcare institutions should create a space for support and continuous care for professionals, recognizing that, in dealing with the pain of others, they may also be dealing with their own pain [79]. In terms of research, studies on the subject can expand the understanding of grief not only from the perspective of bereaved family members but also by considering the experience of healthcare professionals. This is particularly relevant in crisis situations, such as that experienced during the pandemic, where the emotional impact was profound and the grief responses of healthcare workers deserve to be analyzed in their complexity. Research needs to go beyond simply analyzing traumatic responses and seek to understand the different forms of interaction between experiencing and avoiding grief, as well as the individual and social factors that mediate this experience [58,80,81,82,83].

Therefore, it is necessary for healthcare service managers to adopt a proactive stance in recognizing the potential for emotional responses to grief in crisis scenarios. This involves implementing strategies to prepare both professionals and institutions to deal with the complex emotions involved in the experience of loss. Healthcare institutions, in turn, must be attentive to the grief practices that prevail within their organizational culture, identifying any problematic practices and promoting interventions that favor a healthier and more welcoming work environment for healthcare professionals. In addition, the failure to recognize professional grief leads to numerous consequences in terms of emotional exhaustion, burnout syndrome, and depressive states, which affect the mental health of professionals and the quality of care provided. Encouraging further research on institutional culture is also a necessary step towards improving care practices [58,84]. Ultimately, the findings underscore the need for occupational mental health policies that move beyond an exclusive focus on burnout and stress to explicitly include grief, bereavement, and moral injury as legitimate occupational hazards in healthcare settings. Pandemic-related grief was not an exceptional or individual phenomenon, but a collective and systemic experience, requiring policy responses at institutional and national levels. In this vein, policies that normalize emotional expression and grief within healthcare organizations may reduce stigma, prevent chronic psychological distress, and promote workforce sustainability.

## 5. Conclusions

The study’s findings highlight the effects of loss and grief experiences among healthcare professionals during the COVID-19 pandemic. Faced with an unexpected and chaotic scenario, a large number of losses, isolation, and contact restrictions caused a constellation of negative emotional repercussions, such as stress, feelings of fear, depression, sadness, and anger. The experience of grief was intensified by the peculiarities that permeated the death process resulting from the pandemic context, from the circumstances of illness and loss to the modification of farewell moments. In the face of these difficulties, professionals needed to invest in self-care and self-development, strengthen support networks, and work collaboratively. In this vein, further research is recommended to develop and evaluate interventions that facilitate grief coping for healthcare professionals when confronted with death in diverse contexts, especially during times of crisis. At the same time, it is necessary to promote self-care strategies, death literacy, and foster moments and spaces within healthcare organizations to discuss and process the feelings that emerge from these experiences of loss, with appropriate targeted and specialized emotional and psychological support in this area, as well as emphasizing the importance of teamwork as a protective factor.

## Figures and Tables

**Table 1:** Description of professionals (N = 24).

Profession	Age	Gender	Ethnicity	Work Context
1. Nurse	33	Female	White	Inpatient unit
2. Nurse	32	Female	White	Inpatient unit
3. Psychologist	30	Male	White	Inpatient unit
4. Nurse	28	Male	White	Inpatient unit
5. Nurse	31	Female	White	Intensive Care Unit
6. Nurse	32	Female	White	Primary Health Care Unit
7. Nurse	54	Male	White	Intensive Care Unit
8. Nurse	28	Female	White	Intensive Care Unit
9. Psychologist	27	Male	White	Primary Health Care Unit
10. Psychologist	32	Female	White	Intensive Care Unit
11. Nurse	32	Female	White	Intensive Care Unit
12. Nurse	25	Female	White	Inpatient unit
13. Nurse	29	Female	White	Inpatient unit
14. Nurse	28	Male	White	Intensive Care Unit
15. Nurse	27	Male	White	Intensive Care Unit
16. Psychologist	35	Female	White	Primary Health Care Unit
17. Nurse	33	Female	White	Primary Health Care Unit
18. Physician	45	Male	White	Inpatient unit
19. Physician	59	Male	Non-white	Intensive Care Unit
20. Physician	44	Male	White	Intensive Care Unit
21. Psychologist	29	Female	White	Intensive Care Unit
22. Psychologist	48	Female	White	Inpatient unit
23. Nurse	31	Male	Non-white	Inpatient unit
24. Physician	44	Female	White	Inpatient unit

## Data Availability

The datasets used and/or analyzed during the current study are available from the corresponding author upon reasonable request.

## Appendix A. Semi-Structured Interview Guide

1. What was it like for you working on the front lines during the COVID-19 pandemic?
1.1. What were the main challenges encountered?
1.2. What were the facilitating aspects?
2. How did the frequent deaths and illnesses of patients, family members, or colleagues during the pandemic affect their well-being and quality of life?
3. What experiences have most marked and transformed your perspective on patient/family care in the face of visitation restrictions and the impossibility of a final goodbye?
4. What lessons were learned from the COVID-19 pandemic, and what actions would be taken differently today? What would change and why?
5. Is there anything else you’d like to share, or that you consider important?
